# A nearest-neighbors network model for sequence data reveals new insight into genotype distribution of a pathogen

**DOI:** 10.1186/s12859-018-2453-2

**Published:** 2018-12-12

**Authors:** Helen N. Catanese, Kelly A. Brayton, Assefaw H. Gebremedhin

**Affiliations:** 1 School of Electrical Engineering and Computer Science, Washington State University, Pullman, WA USA; 2 Department of Veterinary Microbiology and Pathology, Washington State University, Pullman, WA USA

**Keywords:** Sequence similarity network, Network analysis, Centrality, Clustering, *Anaplasma marginale* Msp1a, GroEL

## Abstract

**Background:**

Sequence similarity networks are useful for classifying and characterizing biologically important proteins. Threshold-based approaches to similarity network construction using exact distance measures are prohibitively slow to compute and rely on the difficult task of selecting an appropriate threshold, while similarity networks based on approximate distance calculations compromise useful structural information.

**Results:**

We present an alternative network representation for a set of sequence data that overcomes these drawbacks. In our model, called the Directed Weighted All Nearest Neighbors (DiWANN) network, each sequence is represented by a node and is connected via a directed edge to *only* the closest sequence, or sequences in the case of ties, in the dataset.

Our contributions span several aspects. Specifically, we: (i) Apply an all nearest neighbors network model to protein sequence data from three different applications and examine the structural properties of the networks; (ii) Compare the model against threshold-based networks to validate their semantic equivalence, and demonstrate the relative advantages the model offers; (iii) Demonstrate the model’s resilience to missing sequences; and (iv) Develop an efficient algorithm for constructing a DiWANN network from a set of sequences.

We find that the DiWANN network representation attains similar semantic properties to threshold-based graphs, while avoiding weaknesses of both high and low threshold graphs. Additionally, we find that approximate distance networks, using BLAST bitscores in place of exact edit distances, can cause significant loss of structural information. We show that the proposed DiWANN network construction algorithm provides a fourfold speedup over a standard threshold based approach to network construction. We also identify a relationship between the centrality of a sequence in a similarity network of an *Anaplasma marginale* short sequence repeat dataset and how broadly that sequence is dispersed geographically.

**Conclusion:**

We demonstrate that using approximate distance measures to rapidly construct similarity networks may lead to significant deficiencies in the structure of that network in terms centrality and clustering analyses. We present a new network representation that maintains the structural semantics of threshold-based networks while increasing connectedness, and an algorithm for constructing the network using exact distance measures in a fraction of the time it would take to build a threshold-based equivalent.

## Background

The dramatic expansion of sequence data in the past few decades has motivated a host of new and improved analytic tools and models to organize information and enable generation of meaningful hypotheses and insights. Networks are one tool to this end, and have found many applications in bioinformatics. One network model with such applications is the protein homology network, in which sequences are connected based on their functional homology. Such networks enable, among other tasks, sequence identity clustering [1]. The subset of these protein homology networks for which edges are built only in terms of sequence similarity are called *sequence similarity networks* (SSN) [2], and these are the class of networks discussed in this work.

SSNs are networks in which nodes are sequences and edges show the distance (dissimilarity) between a pair of sequences. Unlike protein interaction networks, or annotated similarity networks, the distance between sequences is the only feature used to determine whether or not an edge will be present. These networks can be used as substitutes for multiple sequence alignments and phylogenetic trees and have been found to correlate well with functional relationships [2]. SSNs also offer a number of analytic capabilities not attainable with multiple sequence alignment or phylogenetic trees. They can be used as a framework for identifying complex relationships within large sets of proteins, and they lend themselves to different kinds of analytics and visualizations, thanks to the large number of tools that already exist for networks. Centrality (node importance) analysis is one example of an analytic tool enabled by SSNs. Clustering, often for identifying homologous proteins, is another important structure discovery tool.

In this work we present a new variant of SSN, called the **Di**rected **W**eighted **A**ll **N**earest **N**eighbors (DiWANN) network, and an efficient sequential algorithm for constructing it from a given sequence dataset. In the model each sequence *s* is represented by a node *n*_*s*_, and the node *n*_*s*_ is connected via a directed edge to a node *n*_*t*_ that corresponds to a sequence *t* that is the *closest* in distance to the sequence *s* among all sequences in the dataset. In the case where multiple sequences tie for being closest to the sequence *s*, all of the edges are kept. The weights on edges correspond to distances.

We apply this model to analyze protein sequences drawn from three different applications: genotoype analysis, inter-species same protein analysis, and interspecies different protein analysis. We show that the model is faster to compute than an all-to-all distance matrix, enables analytic algorithms such as clustering and centrality analysis with comparable accuracy more quickly, and is resilient to missing data. Neighborhood graphs^1^ more generally have previously been used in bioinformatics for tasks such as inferring missing genotypes [3] and protein ranking [4]. However they have not been used to model and analyze sequence similarity prior to this study.

### Related work and preliminary concepts

#### Other network models in bioinformatics

There are several types of networks other than SSNs used in bioinformatics. Protein–protein interaction networks designate each protein as a node and connect two nodes by an edge whenever there is a corresponding signal pathway [5]. Such networks are the foundation for many applications, including ProteinRank, which identifies protein functions using centrality analysis [6]. Gene regulatory networks are bipartite networks where one vertex set corresponds to genes, the other vertex set corresponds to regulatory proteins, and an edge shows where a regulatory protein acts on a gene [7]. Gene co-expression networks build an edge between pairs of genes based on whether they are co-expressed across multiple organisms [8]. Such networks enable gene co-expression clustering [9] as well as microarray de-noising through centrality analysis [10].

#### Similarity/distance measures

In order to build a network from a set of data where there is no inherent concept of relation, some similarity or distance measure must be used. Many distance measures exist for sets of numeric data, including Euclidian distance and Cosine similarity. For set data, boolean distance measures like Jaccard distance and Hamming distance [11] are commonly used. Jaccard distance is the ratio of the size of the intersection to that of the union of the two sets, while Hamming distance counts the positions at which the two sets differ. For string data, such as protein and DNA sequences, a straightforward option is Levenshtein distance, or edit distance, which is the minimum number of insertions, deletions and mutations needed to convert one string to another [12]. Other distance metrics on strings include Hamming distance, which is faster to compute and handles replacements well but insertions and deletions poorly, and variants of the Needleman-Wunsch [13] and Smith-Waterman [14] alignment algorithms. Both of the latter algorithms use dynamic programming to find the optimal way of aligning two sequences from which distance can be inferred. The use of a scoring matrix can also weight these alignment scores to be more representative of real-world mutation probabilities.

A shared weakness of the pairwise alignment-based and the Levenshtein distance-based methods for exact distance calculation is that they take quadratic time in sequence lengths, which can be prohibitively costly. Faster heuristic (approximate distance) approaches such as FASTA [15] or BLAST [16] and its variants have filled the gap in some cases. However, the similarity scores, bitscores and e-value provided by BLAST were not designed to be used in this way, and for some applications such heuristics have been shown to perform poorly [17–19].

A very different approach to measuring distances on sequences is presented in [20], where strings are represented as time series data, with each mutation, insertion or deletion assigned a particular positive or negative value, so that numeric distance measures could be applied. While this measure is computationally faster, it is sensitive to alphabet ordering, and modifications of different characters entail varying degrees of effect on the distances computed, restricting its potential use to only small alphabets such as DNA. Another way to approximate distance within a fixed bound is to use n-grams, or overlapping substrings of length *n* of a sequence. The idea is that if the number of the n-grams that mismatch between two strings is *d*, then the edit distance between those strings is at most *nd*. This method has been used for pruning string similarity joins [21], however as an approximate distance measure it provides a very loose bound on similarity.

#### Neighborhood network models and algorithms

Many methods exist for generating a similarity network from a set of data using some similarity or distance measure on the data and a threshold. Typically the selection of threshold is achieved through trial and error. While methods for automating the threshold selection have also been proposed [22], the methods do not eliminate the need for all-to-all distance calculations, making them especially unsuitable for costly distance measures.

The class of neighborhood networks is another alternative. In general neighborhood networks rely on finding for every object in the dataset a neighborhood, or set of data points closely related to the object. Edges are then built to connect the object to all or a subset of its neighborhood. One common example of this is the k-nearest neighbors graph, or kNN graph [23]. For this model, a similarity or distance measure is used to find the *k*, where *k*≥1 is a specified constant, nearest neighbors of each data point which are then connected to the data point via network edges. If ties are present, they are typically broken randomly. The brute force approach to this problem, which first computes all pairwise distances between points and then uses only those below some threshold to construct edges, takes *O*(*n*^2^) time and space, where *n* is the number of data points.

A variety of more efficient solutions for kNN network construction exist, for both the cases where the underlying kNN problem is solved optimally [24–29] and where it is solved approximately [30–33]. However, many of these methods assume a numeric feature space, and thus cannot be applied directly to sequence data. One way of generating the optimal KNN solution for generic distance measures is preindexing [34], although the work demonstrated only empirical runtime reductions, and distances were computed between dictionary words, which are very short compared to biological sequences. NN-Descent is an example of an inexact solution that also generalizes to any distance metric [35]. The method iteratively improves on an existing approximate kNN network, however it does not specifically optimize on number of distance calculations, and may thus be a poor fit for more expensive measures like edit distance.

None of these algorithms are tie-inclusive, in the sense that if two (or more) objects are equidistant from an object in question, one (or more) of the potential edges may be arbitrarily excluded from being in the graph.

An alternative to this approach is the *all nearest neighbors* (ANN) network, in which an object is connected to only its *nearest* neighbor, or neighbors in the presence of ties, among the objects in the dataset. In contexts where the distance metric makes ties unlikely, whether or not ties are included is not a major concern. However, with discrete measures of distance like edit distance, where ties are likely, excluding ties can lead to missing important structural information. Additionally, it is not typically clear what values of *k* in a kNN model will be appropriate for a given dataset, and the selection of *k* is susceptible to some of the same difficulties as in threshold selection. In light of these facts, this work focuses on a variant of the ANN model.

Most existing ANN algorithms, some of which are modifications of kNN algorithms discussed previously [24, 25] as well as others [36], are designed solely for numeric space. We are not aware of any prior ANN algorithm specifically designed for string distance measures, and only very few solutions exist for generic distance measures. These methods typically use a tree indexing structure to partition the search space [37, 38], although they only offer average case runtime improvements. An approximate solution proposed in [39] improves worst case runtimes with some probability of errors.

## Methods

### Structural analysis

To test the efficacy of the DiWANN network model and its semantic similarity to threshold based networks, we used three sets of protein sequence data representing three different applications: genotype analysis, inter-species same protein analysis, and inter-species different protein analysis.

The first dataset is composed of 284 *Anaplasma marginale* short sequence repeats (SSRs) from the msp1 *α* gene, each consisting of roughly 28 amino acids, as compiled in [40]. SSRs are a type of satellite DNA in which a pattern occurs two or more times. They can be found in coding regions of the genome, and can occur in genes encoding highly variable surface proteins. In these cases, the SSRs are useful for genotyping, or genetically distinguishing one strain from another.

The second dataset includes sequences of the chaperonin GroEL, a molecular chaperone of the hsp60 family that functions to help proteins fold properly [41]. The dataset includes 812 unique protein sequences from 462 species and 177 genera, compiled from GenBank. These sequences range from 550 to 600 amino acids. We collected 10,000 GroEL sequences, however, in this set there were only 3,077 different sequences. We chose to filter out sequences that occurred only once in the dataset, to keep the experiment time short and reduce noise from outliers. This left us with 812 unique sequences.

The final dataset is the gold standard proteins from [42], with confirmed ground truth labels from five protein superfamilies. The sequences vary widely in length from 100 to over 700 amino acids. We used a subset of the data that had high quality labels for both a protein’s family and superfamily, as some sequences were labeled only with a superfamily. This subset includes 852 sequences. This dataset demonstrates how the models handle more diverse sequences, and includes labels for functional groups (enzyme families).

For each dataset, we generated several exact threshold based networks from which one was chosen for further analysis. We generated a single DiWANN network since there is no associated thresholding concept in the DiWANN model. We compared these exact distance networks against a threshold based network generated via a faster approximate distance metric. The comparison is done in terms of both runtime and accuracy of subsequent network analyses (including clustering and centrality analysis).

The distance/similarity metrics used to create the threshold based networks were BLASTP bitscore, BLASTP similarity score, Needleman-Wunsch alignment score and Levenshtein distance. For similarity metrics, we show thresholds in terms of distance from the maximum similarity, for readability. The inclusion of threshold-based networks using both edit distance and alignment score to define edges is to account for potential loss of accuracy in our networks from using edit distance (a less biologically accurate distance metric). While a DiWANN network could be created using a different metric, the algorithm we propose relies on properties that weighted alignment scores do not satisfy, as described in more detail in the Algorithm section. So instead, we attempt to demonstrate the practical comparability of the measures, at least for our datasets.

While other fast approximate nearest neighbor methods, such as Flann [43] exist, they assume that a full distance matrix is given. Because of this they are not suitable (efficient) for cases where calculating the distance matrix itself is the primary cost for generating the network. Therefore, we do not compare against such methods.

#### Basic properties

In a corresponding subsection in the Results section, we present visualizations of the three network types—exact threshold based, inexact threshold based and DiWANN—using an implementation of the force directed layout algorithm [44] from the igraph package [45]. We also give details on the structural differences between networks in terms of connectedness, sparsity and other properties. For this analysis we focused on the *A. marginale* SSR dataset; we note that similar patterns in terms of connectedness and sparsity held for all three sets of data. We present the basic structural properties for the other datasets in the Communities section as well.

#### Centrality

Under this analysis, we identify the most central nodes on each of the three network variants, study how they compare to each other, and see their relationship to other sequence properties. For the analysis we used PageRank centrality, but we note that similar behaviors were observed using betweenness centrality as well. (A detailed review of the applications of PageRank in bioinformatics and other fields is available in [46].) We created visualizations to reveal which nodes are the most central in these networks. For the *A. marginale* SSR dataset, we also present a map that shows how the sequences that were found to be the most central in the network are distributed geographically. In this context, geographic dispersion is defined in terms of the number of unique countries in which a sequence had been recorded.

#### Communities

Under this category, we investigated the community structure in the two labeled datasets, GroEL and gold standard. For threshold based networks, we began with the lowest threshold value producing an average degree above one and continued up to the threshold beyond which clustering results no longer improved.

We calculated the precision and recall for all clusters of significant size (more than 2 members) at two levels of label granularity. To cluster the undirected networks, we used the Louvain algorithm for community detection [47], which has been found in practice to be among the best clustering methods in terms of maximizing modularity. For the directed networks (DiWANN), we also used the Louvain algorithm, treating the graph as undirected for clustering purposes.

We note that some GroEL samples were found across multiple species, and as a result, some samples had multiple labels while each sequence can only be assigned to a single cluster. This led to a maximum recall of less than one. However, this situation was fairly uncommon in the dataset, and typically only occurred at the species (rather than genus) level.

### Resilience to missing data

One potential concern with a new network model is how well it responds to an incomplete dataset when compared with its alternatives. To compare the resiliency to missing data of the DiWANN network against the threshold based networks, we generated five sample datasets from the GroEL sequences, each with a random selection of 60% of the original data. From each sample, we generated a threshold network and a DiWANN network. The clustering precision and recall of these reduced networks, along with some basic structural properties, were compared to the full version of the network to determine how well structure was maintained in the “reduced” networks.

Additionally, we wanted to examine the structural changes to the DiWANN network as more data are removed, as the proportional increase in high weight (weak) to low weight (strong) edges could potentially result in connections that are not necessarily meaningful in practice. To this end, we generated an additional set of five random networks with only 20% of the original data. The edge weight distributions were then plotted for comparison between the full, the 60% and the 20% networks, along with the mean and maximum edge weights for each.

### DiWANN network model and construction algorithm

**The Model.** As noted earlier, a threshold-based approach to network modeling and construction has disadvantages and weaknesses. Specifically, if the distance threshold is set too low, the model can miss important relationships between proteins and more nodes will be left as singletons with no connections. If the threshold is set too high, the graph can become too dense to meaningfully work with and analyze.

In sharp contrast, in the DiWANN network, each sequence (node) is connected to only the closest neighbor(s) among the other sequences in the dataset, and connected from sequences to which it is a closest neighbor in the dataset. This structure sounds simpler than it is. For example, all outgoing edges from a node necessarily have the same weight, whereas incoming edges to a node can have different weights. Additionally, the out-degree of each node is at least one, whereas no statement can be made on the in-degree of a node.

**Fig. 1:**
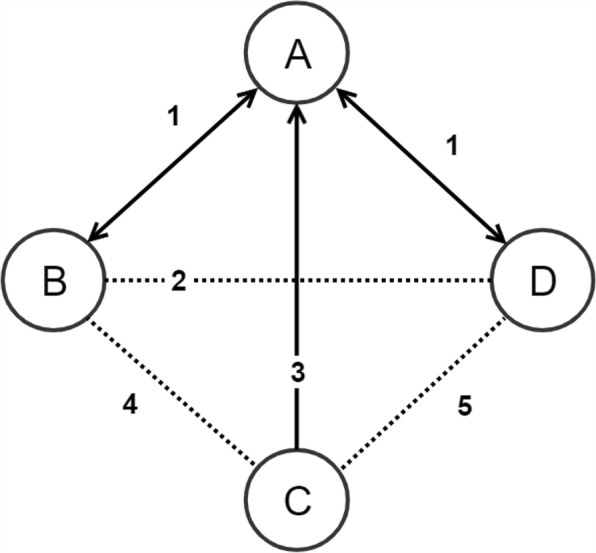
An example showing how DiWANN nodes connect. The example has four nodes, A–D, corresponding to sequences. Weights along the lines show absolute edit distances. Solid lines indicate edges that would be present in the DiWANN graph, while dotted lines show relationships where there would be no edges. The DiWANN graph is structurally different from any threshold-based distance graph


Figure 1 illustrates how DiWANN graph connections are defined. The example shows four sequences A, B, C and D, along with the edit distances between every pair of them. From sequence A’s perspective, sequences B and D, both of which are at distance 1 from A, are its closest neighbors. Therefore, node A is connected via a directed edge of weight 1 to node B and similarly to node D. Likewise, to both sequences B and D, sequence A (at distance 1) is the closest neighbor. Therefore, there is a directed edge of weight 1 from node B to node A and from node D to node A. For sequence C, the closest neighbor, at distance 3, is sequence A. Therefore there is an edge of weight 3 from node C to node A. Note that this extremely simple example still illustrates the case where the in-degree of a node can be zero (C), and the case where the out-degree can exceed 1 (A).

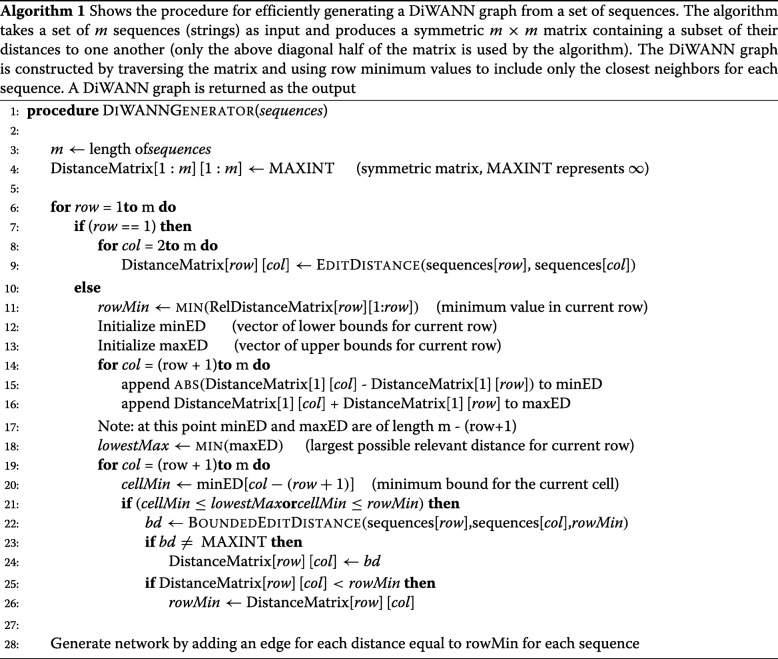


**The construction algorithm.** The DiWANN representation is a succinct summary of the dataset, in the sense that it captures the structural skeleton of the similarity relationships among the sequences, while maintaining connectivity and allowing for analysis that would be meaningful for the original dataset. The formulation naturally lends itself to a more efficient method of generation than producing a pairwise distance matrix for all sequences. The method we present here uses a pruning technique to avoid costly distance calculations in cases where they are not needed. In practice, we found this method to reduce the number of computations and overall time by more than half on the three datasets we considered, as detailed in the Results section.

The algorithm is relatively simple, and relies on a few key features of the DiWANN graph representation to a) prune out the distance calculations that are not needed and b) to bound the calculations that are needed. The procedure is outlined in Algorithm 1. It takes as input a set of *m* sequences and produces an *m*×*m* distance matrix, which is used to generate the DiWANN graph. The algorithm works with only the upper diagonal half of the matrix, and ignores the diagonal and the other half. We describe the algorithm in terms of the *m*×*m* matrix for conceptual simplicity; otherwise in practice the algorithm can easily be implemented with sparse data structures for space efficiency and scalability.

The algorithm begins by initializing each entry of the *m*×*m* matrix to infinity (a sufficiently large number). Next, the matrix is filled out row by row. The entire first row is computed to be used in the pruning phase for subsequent rows.

To prune distance calculations for the remaining rows, the following bounds are used. Assuming the sequence in the first row is *S*_1_ and the distance in question is from sequence *S*_2_ to sequence *S*_3_, the distance lies in the following range:

|*dist*(*S*_1_,*S*_2_)−*dist*(*S*_1_,*S*_3_)|≤|*dist*(*S*_2_,*S*_3_)|≤|*dist*(*S*_1_,*S*_2_)+*dist*(*S*_1_,*S*_3_)|

This property is due to the triangle inequality. Lines 11-21 in Algorithm 1 show the “pruning” optimization, where the value for each cell in a given row is either computed or skipped. In line 21, the distance computation will be skipped if there is some smaller value upcoming in the row based on upper bounds, or if there is already a lower known value. The vectors minED and maxED store a lower and an upper bound for the not-yet-computed distance entries in a row, based on the triangle inequality. The values in maxED are used to compute *lowestMax*, the smallest upper bound for the row, while minED provides the lower bound for pruning entries in a row. The variable *rowMin* tracks a running minimum value for the entire current entry.

**Fig. 2:**
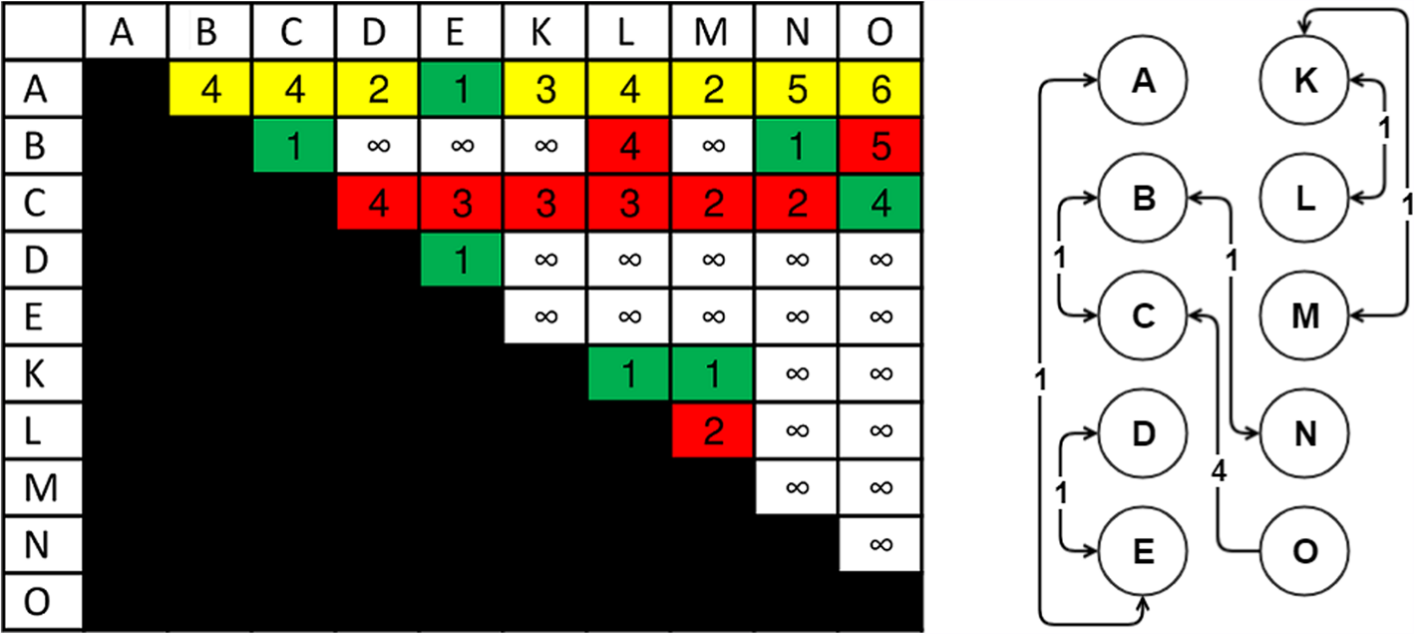
An example illustrating the workings of the DiWANN network construction algorithm. To the left is the distance matrix produced by Algorithm 1, and to the right is the DiWANN graph constructed using this distance matrix. The example has 10 sequences drawn from the *A. marginale* SSRs. Because the distance matrix is symmetric, Algorithm 1 uses only its upper diagonal half, while the unused portion is in black. The first row of the matrix, which must always be computed, is shown in yellow. Every cell in which a distance is computed but is not used in building the DiWANN graph is shown in red. A cell in which a distance is pruned because it wouldn’t result in an edge in the DiWANN graph is shown with entry of infinity. All other non-infinite cell values, shown in green, correspond to edges in the graph. For each sequence, A-O, an outgoing edge is added to the sequence (sequences) that is (are) at the minimum distance from itself (corresponds to *rowMin* at the end of a row computation in Algorithm 1). Note that the edge from node O is not bidirectional

Lines 22-24 correspond to the “bounding” optimization. Here if the distance between the relevant sequences has not been pruned, the computation is done using a bounded Levenshtein distance calculation via the function BOUNDEDEDITDISTANCE (line 22). BOUNDEDEDITDISTANCE takes as parameter two sequences as well as a distance bound, and it either (i) returns the edit distance between the sequences, if that values is at or below the bound, or (ii) terminates early and returns infinity if the distance would be greater than the bound. Here, the bound is *rowMin*, as defined previously. Fig. 2 illustrates how Algorithm 1 works on an input sample of 10 sequences. The example shows how the distance matrix is built, and how the DiWANN graph is constructed from it.

**Runtime complexity.** Calculating the edit distance (or alignment score) between two sequences each of which is of length *n* takes *O*(*n*^2^) time. To do this for a set of *m* such strings, where there are *m* choose 2 pairs of strings, takes *O*(*n*^2^·*m*^2^) time. This can become problematic where either the length or number of strings is large.

Since the DiWANN model needs to maintain only the minimum distance edges, it allows for the pruning and bounding optimizations as described earlier. The bounding optimization reduces the time complexity of calculating the distance between two strings from *O*(*n*^2^) for the standard method to *O*(*n*·*b*), where *b* is the bound and *n* is the length of a sequence. This reduces the complexity for the overall algorithm to *O*(*n*·*b*·*m*^2^), where *b*≤*n*. The benefit of the pruning optimization is not as easy to quantify, but in the worst case, the complexity remains *O*(*m*^2^); the worst case being when the row computed for bounding is similarly distant from all other sequences. It should however be noted that in the case of protein sequences, the level of dissimilarity needed for the worst case scenario to hold, although dependent on the data in use, is highly unlikely, as related sequences are by definition fairly similar.

## Results

### Structural analysis

The following three parts of this subsection discuss results on the basic structure, centrality and communities of the sequence networks we studied. The parts on basic properties and centrality focus on the *A. marginale* SSR network, which was more cohesive, while the communities section focuses on the GroEL and gold standard datasets, for which we have ground truth labels.

#### Basic properties

The three network types we consider (exact threshold based, inexact threshold based and DiWANN) vary in structure in terms of density, connectedness, centrality and a number of other features. In this section, we break down the differences between these network models.

**Fig. 3:**
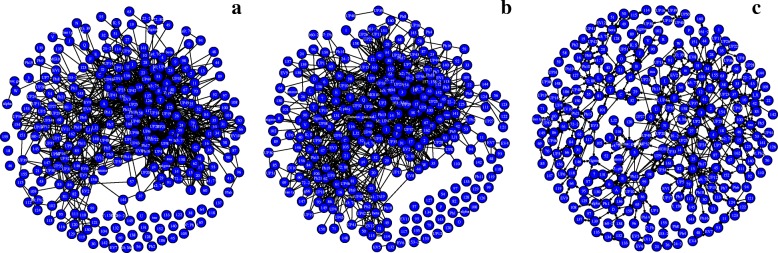
*A. marginale* sequence similarity networks. Subplot **a** shows the inexact similarity network at a 6% difference threshold. Subplot **b** shows an exact distance network at threshold 2. Subplot **c** shows the DiWANN network. All three graphs are for the *A. marginale* SSR data set

**Fig. 4:**
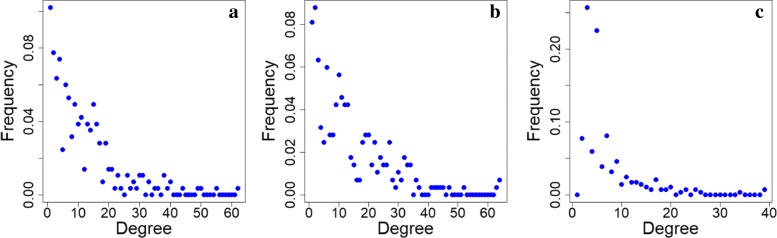
Degree distributions of *A. marginale* sequence similarity networks. This figure shows the degree distributions for each of the SSNs shown in Fig. 3. Subplot **a** shows the degrees of the inexact similarity network at a 6% difference threshold. Subplot **b** shows degrees of the an exact distance network at threshold 2. Subplot **c** shows degrees (combined in and out degree) of the DiWANN network. All three graphs are for the *A. marginale* SSR data set

**Fig. 5:**
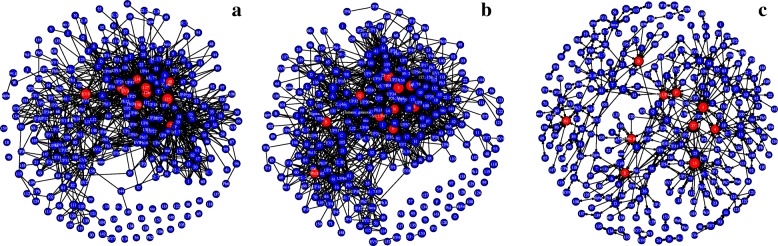
*A. marginale* sequence similarity networks with the most central nodes highlighted. Each figure has been modified to size nodes by their PageRank centrality. The ten most central nodes are highlighted in red. Subplot **a** shows the inexact similarity network at a 6% difference threshold. Subplot **b** shows an exact distance network at threshold 2. Subplot **c** shows the DiWANN network. All three graphs are for the *A. marginale* SSR data set

**Fig. 6:**
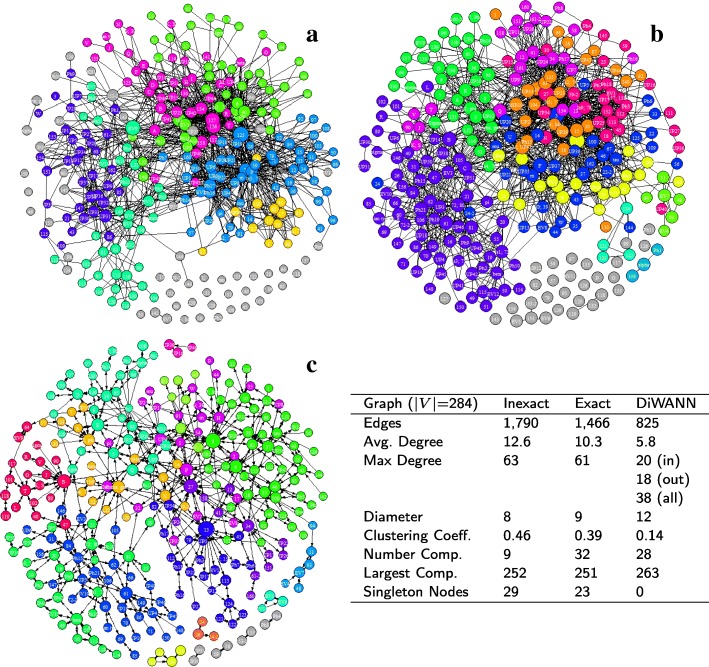
*A. marginale* sequence similarity networks. Subplots **a**-**c** show the inexact (Blast similarity score > 95%), exact (distance ≤ 2) and DiWANN networks for the *A. marginale* SSR data, respectively. The table gives some structural properties for each of these networks. Nodes are sized based on their PageRank centrality, and colored based on their cluster membership using the Louvain community detection algorithm

**Fig. 7:**
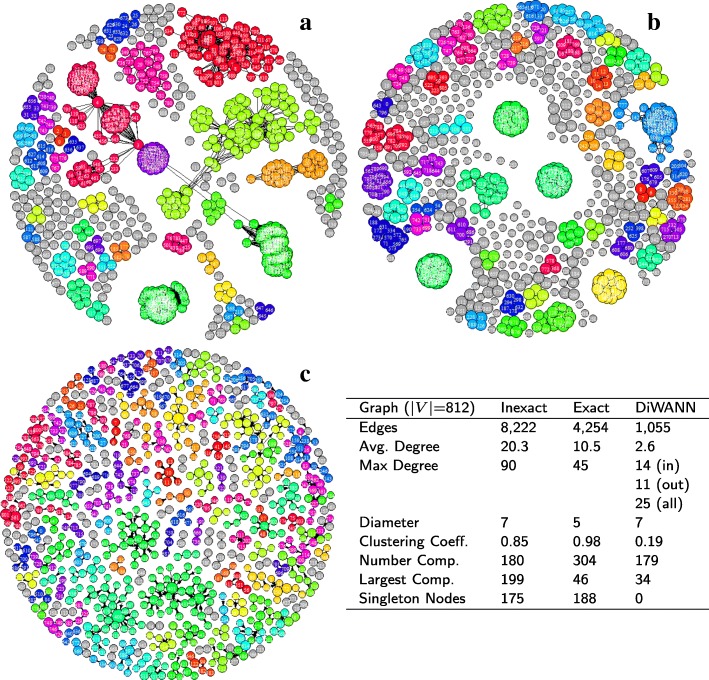
GroEL sequence similarity networks. Subplots **a**-**c** show the inexact (Blast similarity score > 75%), exact (distance ≤ 30) and DiWANN networks, respectively, for the GroEL data. The table gives some structural properties for each of these networks. Nodes are sized based on their PageRank centrality, and colored based on their cluster membership using the Louvain community detection algorithm

**Fig. 8:**
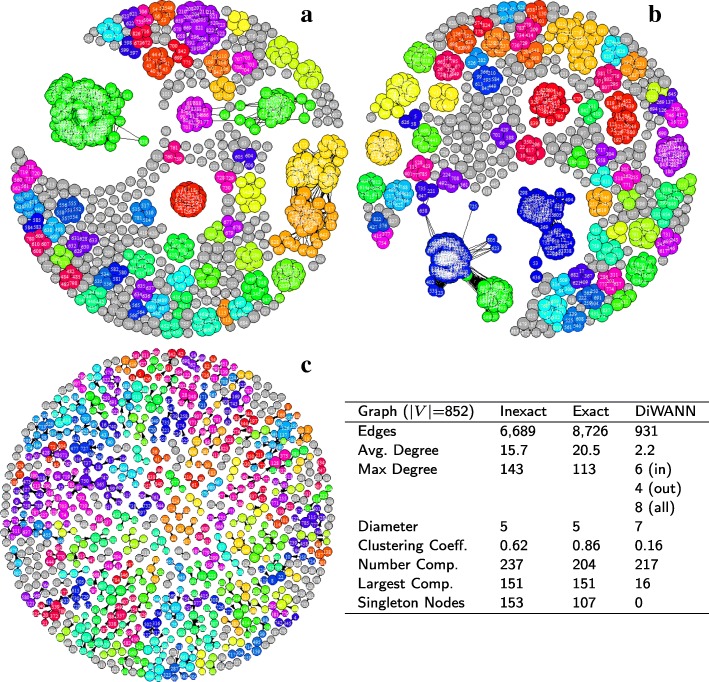
Gold standard sequence similarity networks. Subplots **a**-**c** show the inexact (Blast similarity score > 55%), exact (distance ≤ 150) and DiWANN networks, respectively, for the gold standard data. The table gives some structural properties for each of these networks. Nodes are sized based on their PageRank centrality, and colored based on their cluster membership using the Louvain community dtection algorithm

Figure 3 shows the three network variants for the *A marginale* SSR dataset. It can be seen that both the exact and inexact threshold based networks have a number of singleton nodes which are disconnected from the larger network. Despite this, the threshold based networks are found to be notably denser than the DiWANN network, even at low thresholds. Figure 4 shows the degree distributions of the three networks for the same dataset (*A. marginale* SSRs), which also demonstrates the relative sparsity of the DiWANN network. More details on structural properties of the three network variants on the same dataset is shown in Figs. 5 and 6. The analog of Fig. 6 for the GroEL sequences data is shown in Fig. 7, and the same for the gold standard sequences data is shown in Fig. 8.

From Figs. 3–8, it can be seen that the DiWANN graph merges desirable features of high and low threshold graphs in several relevant ways. In terms of sparsity, it has roughly the same number of edges as the lower threshold graphs. Still, it is either as connected or more connected than the higher threshold graphs.

#### Centrality

The most central nodes were found to be fairly stable across the various exact threshold and DiWANN networks. Among the ten most central nodes for each of these networks, on average about 80% were found to be the same in any two of the exact threshold and DiWANN networks. However, the central nodes for the inexact threshold networks did not appear to be related. The correspondence between the topmost central nodes in these networks and those in the exact distance networks averaged near zero. Figure 5 shows the three *A. marginale* networks with nodes sized by centrality scores (PageRank) and the top ten most central nodes highlighted in red. Figures 7 and 8 show similar results for the GroEL and gold standard datasets, respectively.

**Fig. 9:**
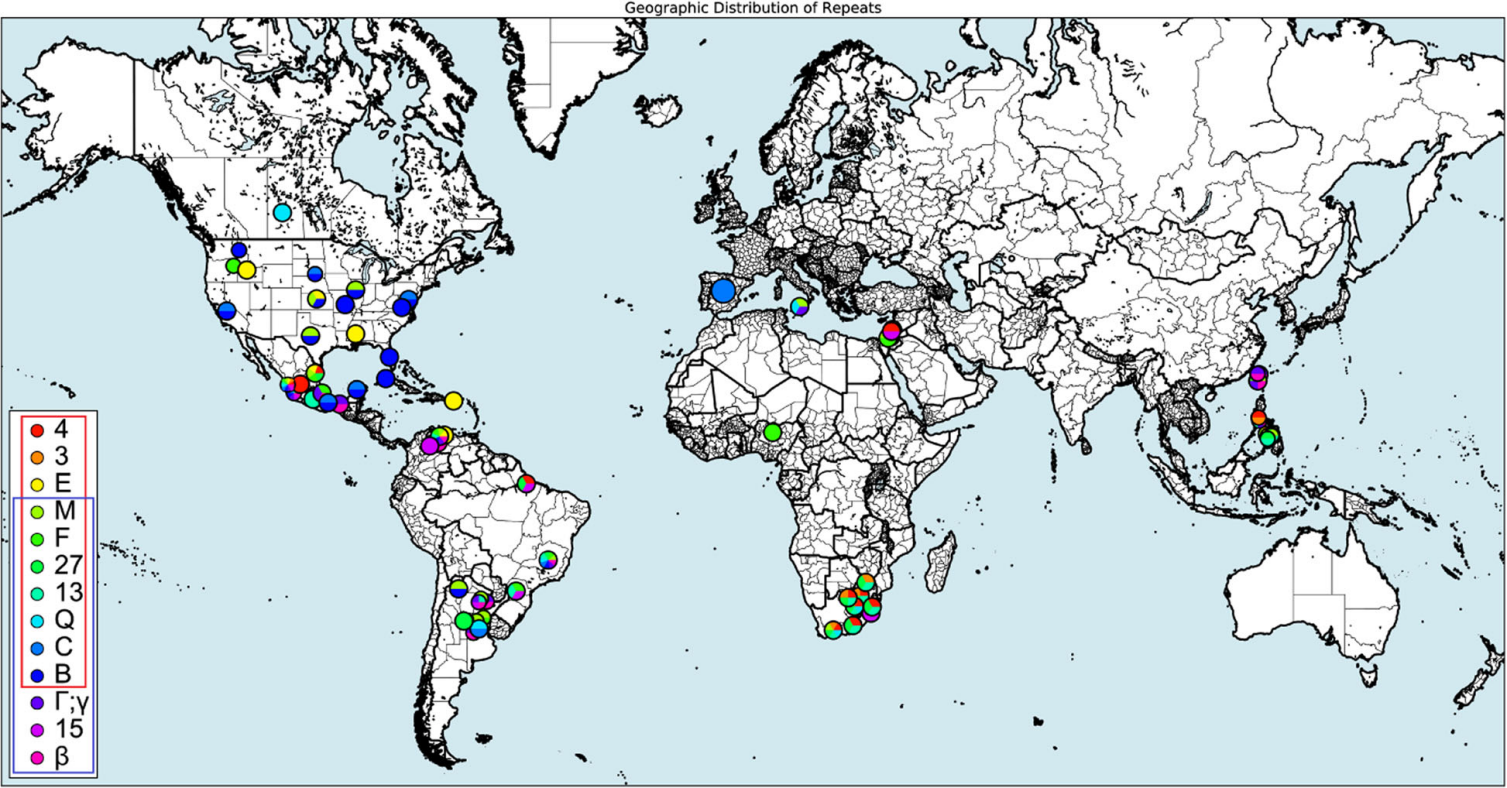
A map of the 10 most common and 10 most central *A. marginale* Msp1a SSRs. This map, generated by RepeatAnalyzer, shows the distribution of the 10 SSRs which appear across the greatest number of countries, as well as those which are most central in the graph representation of the data. In the legend, central SSRs are in the red box, while common SSRs are in the blue box

**Fig. 10:**
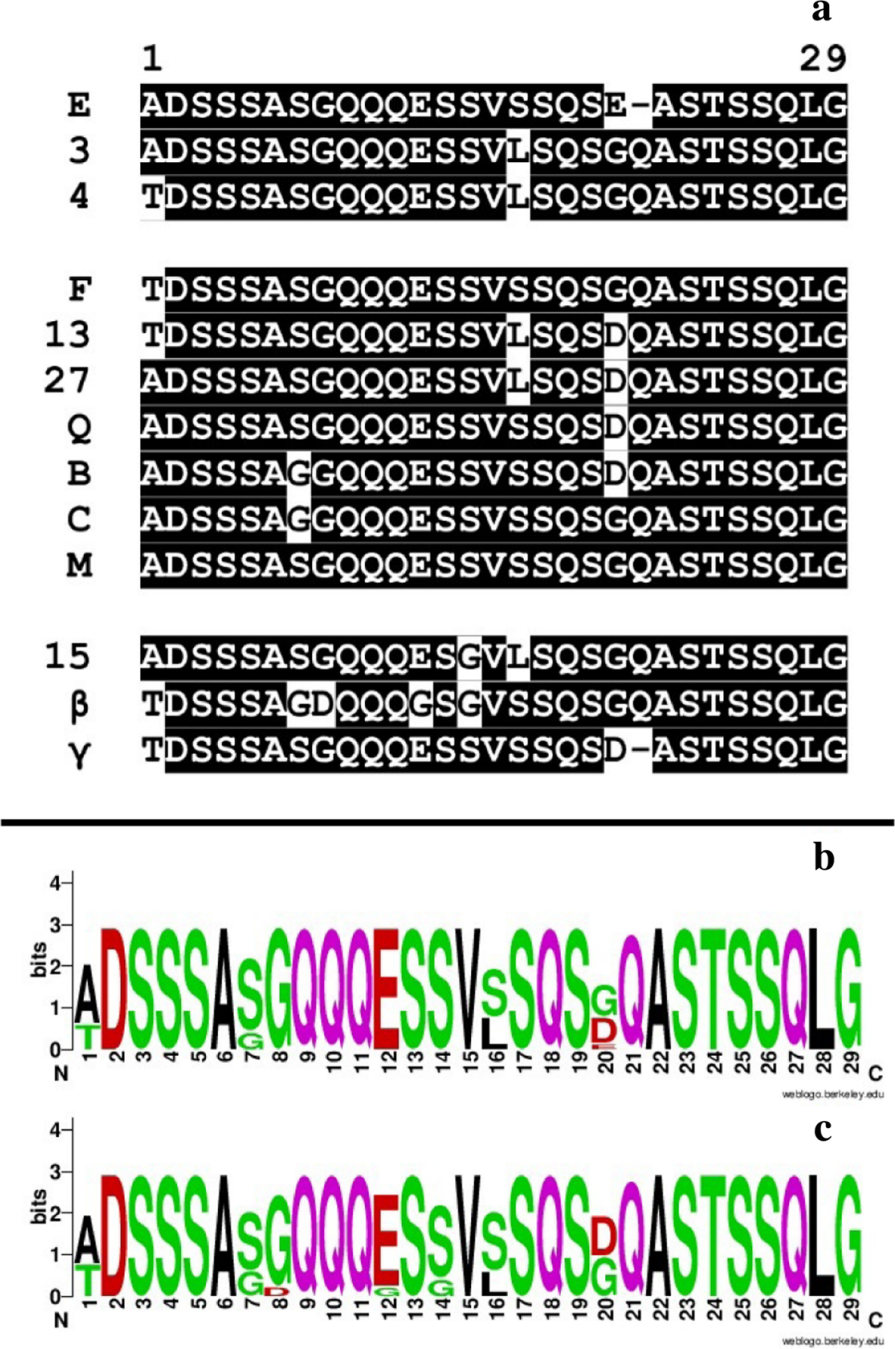
Alignment and logos of the most common and most central SSRs. Panel **a** of this figure shows the alignment for the ten most common (geographically) and ten most central (in the DiWANN network) *A. marginale* SSRs. The top three entries are SSRs which are only common, while the bottom 3 are only central. Those in the middle seven rows belong to both sets. The logo in panel **b** represents the most central SSRs, while the one in panel **c** represents the most common SSRs

It has already been noted that some *A. marginale* Msp1a SSRs, such as M [48], are widely geographically distributed, which we confirmed here. However we have found an additional pattern of interest for these widely dispersed SSRs relating to their centrality. Specifically, those nodes that are most geographically dispersed also tend to be the most central in the network. As shown in Fig. 9, seven out of ten of the most central and most common sequences are the same. This pattern held roughly across each of the exact threshold graphs we worked with, as well as the DiWANN graph, as the central nodes across them were consistent for the most part. Because no such pattern existed for the inexact networks, we suspect that some meaningful structure was lost due to the approximation of distances. Figure 10 shows the alignment of the central and common *A. marginale* sequences, alongside the logo [49] of each.

#### Communities

For the *A. marginale* SSR data, we lack ground truth values for clustering, however the gold standard data are labeled, and for the GroEL data we used genus and species as ground truth labels.

For the GroEL samples the majority of network variants (excluding high threshold BLAST networks) were highly fragmented, having hundreds of connected components (see the table in Fig. 7). This is not unexpected as data were collected from dozens of different species. On these networks, we used the Louvain clustering algorithm to generate groups of samples. For the aforementioned disconnected networks, we found that the clusters corresponded almost exactly along connected component lines.

For the GroEL data we generated clustering results on both the exact and inexact networks over a variety thresholds, as well as for the DiWANN network. Table 1 shows the specific precision and recall values for each network for both genus and species. Overall the exact networks produced strong clusters in terms of both precision and recall compared to the inexact threshold-based networks. Between the threshold based networks and DiWANN, the threshold based networks have higher precision at the cost of both recall and cluster coverage. This demonstrated that a significant level of clustering accuracy is sacrificed when using approximate distance measures, at least for this dataset, and that the DiWANN network performs comparably to threshold based networks, even in some ways better.

**Table 1:** Clustering accuracy for the GroEL networks

Graph (812 nodes)	Th.	Edges	C	|*C*_1_|	|*C*_2_|	|*C*_>2_|	Genus prec.	Genus recall	Species prec.	Species recall
Similarity Score	5%	2886	371	246	122	444	43.0%	21.6%	34.9%	43.0%
	15%	4668	275	175	90	547	38.9%	22.2%	30.3%	40.0%
	25%	8222	182	122	44	646	33.0%	31.8%	22.4%	36.9%
	35%	12,491	81	55	22	735	26.5%	18.6%	17.1%	28.0%
Bitscore (from max)	50	544	623	552	86	174	30.5%	21.7%	24.7%	51.3%
	100	2895	367	243	122	447	42.1%	20.2%	34.0%	42.0%
	200	4576	275	175	86	551	38.7%	21.7%	29.9%	42.1%
	300	9271	183	126	40	646	31.8%	26.2%	22.4%	33.8%
Edit Distance Threshold	8	2139	456	345	128	339	97.3%	33.4%	77.9%	58.3%
	16	2904	391	268	126	418	95.9%	35.3%	72.7%	59.1%
	30	4254	304	188	118	506	90.3%	47.3%	60.6%	63.2%
	42	5023	256	154	90	568	85.0%	51.9%	56.5%	64.4%
	54	6582	206	114	76	622	81.5%	58.3%	50.8%	66.6%
	60	7196	190	99	80	633	80.6%	62.6%	49.3%	66.8%
Needleman-Wunsch (from max score)	100	1780	482	386	110	316	42.2%	4.7%	30.8%	5.8%
	200	4691	280	175	98	539	87.0%	49.7%	59.4%	63.5%
	300	7733	183	96	80	636	79.1%	62.5%	48.0%	66.8%
DiWANN	NA	1055	180	0	118	694	80.4%	43.9%	59.5%	61.8%

This table shows a summary of clustering accuracy for the various GroEL networks. Th. gives the threshold used for a given network, either in number of edits, distance from the maximum similarity score (for bitscore and Needleman-Wunsch) or percent similarity score. C gives the total number of clusters, |*C*_1_| gives the number of nodes in clusters of size 1 (singletons), |*C*_2_| gives the number of nodes in clusters of size 2, and |*C*_>2_| shows the number of nodes in clusters of size 3 and above. For calculating precision and recall, we assume clusters should correspond to the genus and species labels for a given GroEL sequence. Each GroEL sequence is between roughly 550 and 600 amino acids

For the gold standard data, a similar behavior was observed. Networks broke into many connected components, which broke along family and superfamily lines (see Fig. 8). The precision and recall values are shown in Table 2. For these data the recall values were not high, primarily because each superfamily tended to break into many components. Note that while for most of the networks clustering took negligible time, higher threshold networks (above 100) took up to fifteen minutes to run due to their density.

**Table 2:** Clustering accuracy for the gold standard data

Graph (852 nodes)	Th.	Edges	C	|*C*_1_|	|*C*_2_|	|*C*_>2_|	SF prec.	SF recall	Family prec.	Family recall
Similarity Score	15%	1563	427	300	116	432	49.3%	2.2%	35.4%	4.7%
	25%	3057	335	223	88	537	46.4%	3.2%	33.0%	5.1%
	35%	5125	265	169	72	607	42.7%	5.2%	30.6%	7.0%
	45%	6689	239	153	64	631	41.5%	7.1%	30.4%	8.8%
	55%	7433	223	140	64	644	40.7%	6.8%	29.8%	8.1%
Bitscore (from max score)	1000	1808	507	436	64	348	44.3%	3.6%	32.5%	4.1%
	1100	4168	364	293	56	499	42.7%	5.2%	30.7%	5.8%
	1200	5607	300	223	50	575	42.1%	4.7%	30.6%	5.8%
Edit Distance Threshold	50	1134	448	312	122	418	100%	4.2%	99.3%	21.9%
	100	3453	310	192	86	574	100%	8.2%	98.6%	32.4%
	150	8726	206	107	74	671	99.0%	15.5%	95.8%	45.6%
	175	12,966	152	71	54	727	94.6%	25.6%	91.3%	57.7%
	200	18,097	115	48	38	766	93.6%	29.5%	88.3%	60.7%
Needleman-Wunsch (from max score)	2200	1896	638	588	32	232	100%	8.8%	100%	33.5%
	2400	5485	507	450	56	346	100%	17.9%	100%	48.5%
	2600	8231	378	324	38	490	100%	18.8%	100%	51.8%
	2800	15,603	291	228	46	578	100%	30.5%	100%	64.3%
	3000	26,917	243	176	38	638	99.8%	39.7%	94.8%	79.1%
	3200	39,633	165	123	26	670	83.8%	64.2%	68.4%	87.7%
DiWANN	NA	931	218	0	142	710	97.5%	3.5%	92.3%	25.5%

This table shows a summary of clustering accuracy for the gold standard sequence similarity networks. Th. gives the threshold used for a given network, either in number of edits, distance from the maximum similarity score (for bitscore and Needleman-Wunsch) or percent similarity score. C gives the total number of clusters, |*C*_1_| gives the number of nodes in clusters of size 1 (singletons), |*C*_2_| gives the number of nodes in clusters of size 2, and |*C*_>2_| shows the number of nodes in clusters of size 3 and above. For calculating precision and recall, we assume that clusters correspond to the family and superfamily labels provided with the dataset. Sequences vary widely in length between 100 up to over 700 amino acids

### Resilience to missing data

To test the resilience to missing data of the DiWANN network compared to the threshold based networks, we generated five random samples, each containing 60% of the proteins in the GroEL dataset. From these samples, we generated five networks. Table 3 shows the structure and clustering comparison for these reduced networks and their full counterparts. While there are minor variations in precision and recall, overall the reduced networks produced similar qualities of clusters. The reduced DiWANN networks were more significantly altered in terms of structure, as indicated by the variation in clustering coefficient; nonetheless, the clustering results produced from those networks were to a large extent unaffected.

**Table 3:** Structural comparison of networks on subsets of the data (resilience to incomplete data)

	Avg. degree	D	CC	Number Comp.	Largest Comp.	|*C*_1_|	|*C*_>2_|/|*V*|	Genus prec.	Genus recall	Species prec.	Species recall
GroEL Threshold											
Full	10.5	5	0.98	304	46	188	62.3%	90.3%	47.3%	60.6%	63.2%
Sample Avg.	6.2	4	0.97	221	29	150	56.1%	89.7%	46.7%	62.8%	64.5%
Sample 1	6.2	5	0.97	221	30	151	53.8%	90.8%	47.2%	62.2%	65.4%
Sample 2	6.3	4	0.97	229	29	158	52.0%	89.3%	45.4%	62.1%	62.6%
Sample 3	6.5	4	0.98	223	27	152	53.2%	89.2%	45.5%	61.0%	63.0%
Sample 4	6.3	5	0.96	212	32	141	56.7%	87.7%	46.2%	63.4%	64.9%
Sample 5	5.9	4	0.98	218	25	146	58.5%	91.6%	49.4%	65.3%	66.7%
GroEL DiWANN											
Full	2.6	7	0.19	179	34	0	85.4%	80.4%	43.9%	59.5%	61.8%
Sample Avg.	2.7	6	0.41	119	26	0	86.4%	75.8%	51.1%	55.2%	67.8%
Sample 1	2.8	7	0.52	100	33	0	86.4%	73.6%	52.1%	53.4%	68.4%
Sample 2	2.4	5	0.21	111	22	0	85.6%	78.2%	50.4%	56.4%	66.2%
Sample 3	2.8	6	0.58	113	24	0	84.0%	73.6%	52.7%	54.0%	69.2%
Sample 4	2.6	5	0.38	105	23	0	87.3%	77.9%	46.0%	57.9%	67.6%
Sample 5	2.7	7	0.36	105	29	0	88.9%	75.8%	54.1%	54.5%	68.0%

This table shows a comparison of both structure and clustering results for the GroEL dataset for networks generated from a random sample of 60% of the sequences. D denotes diameter, and CC denotes clustering coefficient. Also shown are the number of connected components, and the size of the largest component. |*C*_1_| gives the number of nodes in clusters of size 1 (singletons), and |*C*_>2_|/|*V*| shows the percentage of nodes in a cluster of size 3 or above. The full network is also included for comparison. For the threshold based networks, we use a threshold of 30, which had a good trade-off of precision and recall in the community analysis. The full networks contain 812 nodes, while each reduced network contains 487 nodes

**Fig. 11:**
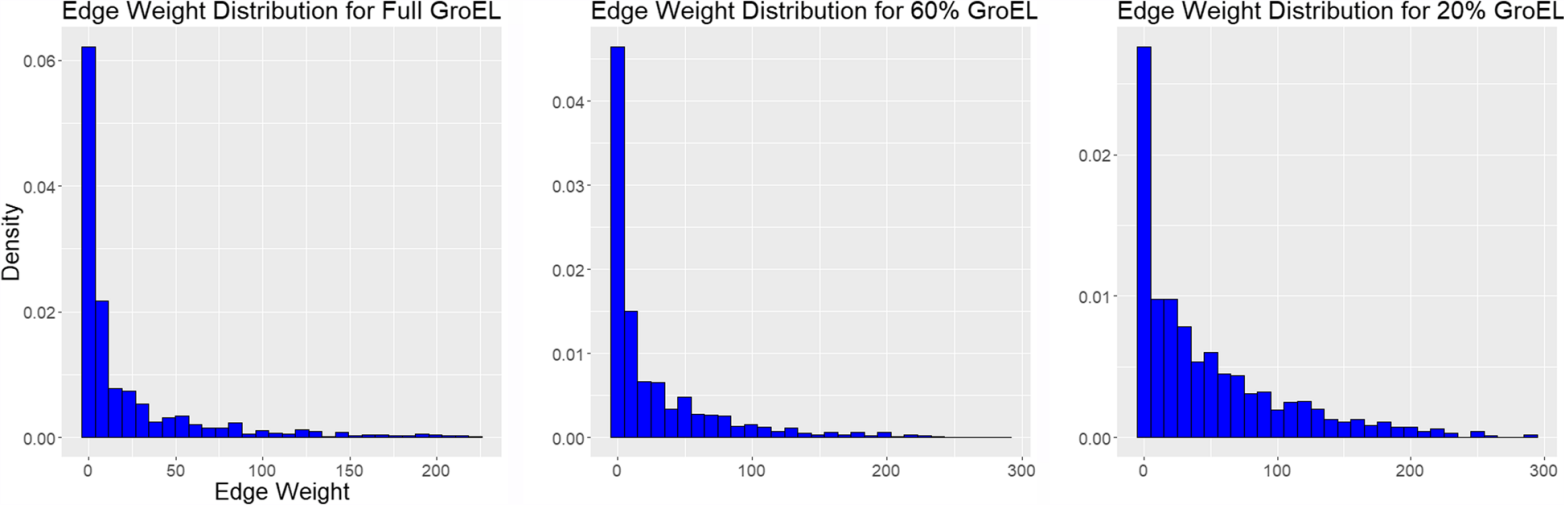
Edge weight distributions for the GroEL dataset. These plots show the distribution of edge weights in the DiWANN networks using the full GroEL data, averages for five 60% samples and averages for five 20% samples. The minimum edge weight for all three networks is 1. For the 20% networks, the median is 4 and the maximum is 223. For the 60% networks, the median is 6 and the maximum is 288. For the full network, the median is 29 and the maximum is 291

Additionally, we wanted to see how the structure of the DiWANN network changes as data are removed. To this end, we plotted the edge weight distributions for the full network, and for the average of two sets of 60% and 20% networks. The weight of an edge can be thought of as a measure of the strength of the connection between those two nodes. Thus, the higher the edge weights in the DiWANN network, the more edges represent weak connections, and the more disparate the sequences in the network. Of course, in a given analysis, it is possible and perhaps even helpful to ignore edges above a certain weight. These plots are meant to give an estimate of how many such weak edges there are at different levels of missing data.The distributions are shown in Fig. 11. As one would expect, as more data are removed, mean edge weight increases, as does maximum, but the power law distribution of edges remains even down to the 20% network. This indicates that while losing data does weaken the average tie strength in the network, the majority of ties are still relatively strong.

### Performance of the graph generation algorithm

In order to assess the runtime gains of our method, we performed an empirical analysis of runtimes of our DiWANN construction algorithm for the three datasets: *A. marginale* SSR data, the GroEL protein sequences from GenBank, and the gold standard protein data.

Table 4 shows the runtime (in seconds) to generate a distance matrix from which the DiWANN graph is trivially built. For two of the three datasets, computing pairwise BLAST scores was significantly faster than computing exact edit distances; in the smallest dataset, the overhead from BLAST made it slightly slower. The DiWANN construction times showed a fourfold improvement over a basic threshold based approach. The method was able to prune approximately 15% of distance calculations for the gold standard data, approximately 26% for the *A. marginale* SSR data, and about 35% of calculations were skipped for the GroEL data. The remainder of the speedup is likely due to the bounding of distance calculations.

**Table 4:** Performance comparison of the network construction algorithms

Method	Msp1a SSR	GroEL	Gold standard
Inexact threshold (seconds)	30.6	2106	211
Exact threshold (seconds)	27.9	100,131	60,434
DiWANN (seconds)	7.4	25,980	20,266

This table shows the times taken to generate the distance matrix for each type of network, which is essentially the time for network generation

## Discussion

As shown in Table 3, the DiWANN network behaved significantly differently than a threshold based network with incomplete data. The average degree of the reduced DiWANN networks remained roughly stable, while in the threshold based case the average degree is reduced in proportion with the node count. However for clustering coefficient in particular, the reduced DiWANN networks varied greatly compared to their full counterpart, as opposed to the threshold based reduced networks, for which clustering coefficient is near constant. This is sensible, insofar as removing a highly connected sequence from the DiWANN network can mean significant structural changes as each node picks a new neighbor(s). In contrast, removing a node from a threshold-based network would simply remove any edge to or from that node, effectively producing a reduced version of the original network. It may be due to this structural flexibility that the number of nodes meaningfully clustered for the reduced DiWANN networks is unaffected relative to the full networks as data are removed at random. Although the precision of the remaining clusters is kept high in the case of the reduced threshold networks, the resulting loss of meaningfully grouped nodes may be an unfavorable trade-off, indicating that in cases where a threshold based approach results in many singleton nodes, DiWANN is a more informative model.

On another point, we find that the DiWANN network performs comparably to threshold based approaches in terms of clustering accuracy, even when a standard network clustering algorithm is used. However, since the DiWANN model is, in a sense, a structural summary of the similarity relationships in the dataset, a specialized clustering algorithm designed to take advantage of the information encoded in the model could give superior clustering results. Design of such a specialized algorithm is an interesting avenue for future work.

In this study, we assumed that edit distance is an analog for similarity. While one could envision using a more biologically significant metric than edit distance, such as weighting edits based on probability, or using an alignment based method with a scoring matrix, such a metric would have caused problems for the symmetry property commonly assumed in distance or similarity measures. This is because there is no guarantee that an edit from one amino acid to another is as likely as an edit in the opposite direction, or that an insertion is as likely as a deletion. More importantly, a weighted measure would disrupt the validity of the triangle inequality (though a much weaker version could still be applied) on which our pruning method is based. Due to these two factors, we have included results for our threshold-based networks using Needleman-Wunsch (NW) alignment scores as well as edit distance, however we only generate the DiWANN network with edit distances. While using NW scores in the DiWANN graph construction does produce better clustering results than using edit distances for our data, the results are sufficiently comparable that we conclude edit distance may be an adequate substitute in many cases.

The relationship between centrality and commonality among SSRs implies a biologically interesting conclusion, but by no means verifies it. Nonetheless, it is of enough interest to mention as an avenue for future inquiry. Because centrality is a property defined in terms of a sequence’s structure and how that relates to the structure of other sequences, while its geographic commonality is a measure of where that sequence occurs, it could be the case that these central/common SSRs were spread in some way and have thus developed many variants over a wide geographic area. Alternatively, these central/common SSRs could be ancestral types that are widely distributed and structurally central for that reason. That most of the SSRs are close in edit distance to each other would give credence to this idea. Interestingly, SSR (Beta) has a closest edit distance of four from any of the other SSRs that are central or common (Fig. 10), arguing that this common SSR is an outlier in the set.

It should be noted that we chose to measure “geographic dispersion” of SSRs in terms of the number of countries in which an SSR occurs, rather than a stricter measure of geographic distance. While we recognize the latter might be more meaningful in some contexts, for many of the samples, the only information available about their location is the country in which they were reported. Further, for some contexts (i.e. trade and national regulatory variations) considering data in terms of countries may elucidate patterns that strict distance would not. However, the same basic methods could be applied using geographic distances as well. It is an open question whether the resulting patterns would be the same, but based on the wide dispersion of common SSRs across distant countries, we suspect it would.

While this paper gives a description of the DiWANN network model and some examples of applications, we can see a number of additional uses for the model. For example, in a set of sequences from different species, clusters composed of multiple species may provide a way of detecting orthologues. The network may also be used as a structural skeleton on which a threshold based network could be built, maintaining the advantages of each. Both of these are interesting avenues for future inquiry.

## Conclusions

Sequence similarity networks are an important tool for understanding the relationships between proteins in a dataset. We have demonstrated that, in some cases, using approximate distance measures such as BLAST similarity scores to generate SSNs rapidly may lead to significant deficiencies in the structure of a network in terms of its central nodes and clusters. We presented a new network model that mitigates some of these weaknesses and can be built in a fraction of the time it would take to construct an exact threshold based network. We showed that the model is resilient to missing sequences, sparse (and thus fast to analyze), and maintains many of the useful structural properties of an exact threshold based network, while achieving a higher level of connectedness. We also showed that protein centrality in a sequence similarity network may be linked to non-structural properties of that sequence, such as its geographic distribution.

## Endnotes

## Abbreviations

DiWANN: Directed weighted all nearest neighbors
KNN: K nearest neighbors
SSN: Sequence similarity network
SSR: Short sequence repeat

## Footnotes

1 We use the terms graphs and networks interchangeably throughout the manuscript.
